# Dietary Xylo-oligosaccharide stimulates intestinal bifidobacteria and lactobacilli but has limited effect on intestinal integrity in rats

**DOI:** 10.1186/1756-0500-7-660

**Published:** 2014-09-19

**Authors:** Ellen Gerd Christensen, Tine Rask Licht, Thomas Dyrmann Leser, Martin Iain Bahl

**Affiliations:** Division of Food Microbiology, National Food Institute, Technical University of Denmark, Mørkhøj Bygade 19, Søborg, DK-2860 Denmark; Chr. Hansen A/S, Bøge Allé 10-12, DK-2970 Hørsholm, Denmark

**Keywords:** Xylooligosaccharides, Bifidobacterium, Gut microbiota, Intestinal integrity

## Abstract

**Background:**

Consumption of prebiotics may modulate gut microbiota, subsequently affecting the bacterial composition, metabolite profile, and human health. Previous studies indicate that also changes in intestinal integrity may occur. In order to explore this further we have investigated the effect of the putative prebiotic xylo-oligosaccharides (XOS) on the gut microbiota and intestinal integrity in male Wistar rats. As changes in intestinal integrity may be related to the expected bifidogenic effect of XOS, we additionally addressed effects of supplementation with a commensal *Bifidobacterium pseudolongum* (BIF) isolated from the same breed of laboratory rats.

**Results:**

Changes in faecal and caecal bacterial composition determined by 16S rRNA gene sequencing and quantitative PCR for selected bacterial groups revealed that the overall bacterial composition did not differ markedly between the control (CON), XOS, and BIF groups, when correcting for multiple comparisons. However as hypothesised, the relative abundance of *Bifidobacterium* spp. was increased in XOS-fed rats as compared to CON in faecal samples after the intervention. Also *Lactobacillus* spp. was increased in both the XOS and BIF groups in caecum content compared to CON. Intestinal permeability determined *in vivo* by FITC-dextran permeability and *in vitro* using extracted caecum water in trans-epithelial resistance (TER) assay showed no effect on intestinal integrity in either the XOS or the BIF groups. However, the expression of occludin, which is part of the tight junction complex, was increased in the XOS group compared to the CON group.

**Conclusions:**

Supplementation with XOS or a commensal *Bifidobacterium pseudolongum* had very limited effects on intestinal integrity in rats as only significant change in expression of a single tight junction protein gene was found for the XOS group.

## Background

The complex microbial community of the gut environment is thought to interact with the host organism and to affect human health [1]. Modulation of the gut microbial composition by consumption of specific substances such as prebiotics and probiotics may therefore affect intestinal and systemic health. Previous studies of the modulatory effect of established prebiotics as well as putative prebiotics have mainly focused on *Bifidobacterium* spp. and *Lactobacillus* spp. in the microbiota [2–5] as these are claimed to have beneficial effects on health [6]. Effects on other bacterial groups, potentially with adverse effects on health, may thus have been overlooked. The development of high-throughput sequencing techniques now makes it feasible to survey the entire microbiota. In addition to determining the effect of pre- and probiotics on the complete gut microbiota, it is important to understand how such effects influence host health. An important marker for health is intestinal integrity, as increased intestinal gut permeability previously has been connected to intestinal disorders including inflammatory bowel diseases and coeliac disease [7, 8]. Gut wall permeability can be determined *in vivo* by examining the permeability of molecules with a defined size, such as FITC-dextran [9] and CrEDTA [10]. In addition, effects on intestinal integrity can be estimated by determining the expression and localization of tight-junction proteins. Effects of gut content on intestinal integrity may also be assessed *in vitro* by examining the effect of metabolites from the community found in e.g. faecal water on trans-epithelial resistance (TER) in epithelial cell monolayers [11, 12].

Several previous studies have examined the effect of prebiotic supplementation on pathogen invasion in animal challenge studies. Prebiotic fructo-oligosaccharides (FOS) and the putative prebiotic xylo-oligosaccharides (XOS) [13] have previously been found to stimulate translocation of *Salmonella* in rats [14, 15] and mice [16]. Here the prebiotics also stimulated increase in *Bifidobacterium* spp. [14, 17] and *Lactobacillus* spp. [14, 15], which are both considered to have a beneficial effect on host health. In connection to this, FOS has been found to increase permeability of CrEDTA in rats, while also stimulating these two groups of bacteria [18]. Also, we have recently shown a trend for an inverse association between the relative abundance of *Bifidobacterium* spp. in human faeces and the effect of faecal water on trans-epithelial resistance (TER) [11]. This however does not necessarily implicate that bifidobacteria or lactobacilli are involved in the observed adverse effects, but the effects could be attributed to other factors, such as changes in non-investigated bacterial groups. The modulation of the microbiota as whole by prebiotics may thus result in adverse effects on the intestinal integrity, which could be due to changes in metabolic outputs of the community. Also *in vitro* studies show that *B. infantis* produce compounds that increase TER [19] and that UV-killed *B. bifidum* and *B. breve* increase TER [20]. Furthermore *in vivo* studies show that bifidobacteria increase intestinal integrity in animal disease models [21, 22]. We hypothesize, that an increase in *Bifidobacterium* spp. caused by e.g. consumption of prebiotics may affect the intestinal integrity indirectly by affecting proliferation and/or metabolic activity of other bacteria, causing conditions that allow increase in *Salmonella* translocation upon challenge. The aim of the present study is thus to determine effects of XOS and commensal bifidobacteria on the gut microbiota and the intestinal integrity in healthy, unchallenged rats using high throughput 16S rRNA gene sequencing quantitative PCR and three different methods to determine intestinal permeability. The study provides new insights into understanding interactions between gut bacterial community composition and intestinal integrity.

## Methods

### Isolation of a commensal *Bifidobacterium*spp. from rats

Faecal samples from Wistar rats were obtained prior to the animal studies from the same facility (Taconic, Lille Skensved, Denmark). Bifidobacteria were isolated from the faecal samples by plating on Bifidus Selective Medium (BSM) agar (Fluka), incubation anaerobically at 37°C for three days, selection for correct colony morphology (pink or dark brown colonies) and verification by PCR using bifidobacteria-specific primers BifF/BifR (Table 1). Universal primers 27 F (5’-AGA GTT TGA TYM TGG CTC AG-3’) and 907R (5’- CCG TCA ATT CMT TTG AGT TT-3’) were used for sequencing. The PCR products obtained with the universal primers were purified by gel-electrophoresis and the 16S rRNA gene partially sequenced using the same primers. Four isolates were found to be identical and have 99.4% sequence homology over 726 bp to *Bifidobacterium pseudolongum* subsp. *globosum* strain JCM 5820 by BLAST search [23]. Since the four isolated strains were identical, we chose a single strain, designated *B. pseudolongum* TR2_39 for this study. Aliquots of TR2_39 (1 ml) were frozen in glycerol and stored at -80°C.

**Table 1:** **Primers used for PCR and quantitative PCR**

Target	Primer	Primer sequence (5’-3’)	Size (bp)	Ref
*Bifidobacterium* spp.	BifF	GCGTGCTTAACACATGCAAGTC	126	[24]
	BifR	CACCCGTTTCCAGGAGCTATT		
*Lactobacillus* spp.	LactoAll_1F	AGCAGTAGGGAATCTTCCA	341	[25, 26]
	LactoAll_1R	CACCGCTACACATGGAG		
*Akkermansia muciniphila*	AM1	CAGCACGTGAAGGTGGGGAC	327	[27]
	AM2	CCTTGCGGTTGGCTTCAGAT		
Universal bacteria	HDA1	ACTCCTACGGGAGGCAGCAGT	200	[28]
	HDA2	GTATTACCGCGGCTGCTGGCAC		
Beta-actin (*Actb*)	ACTB_A	CACCCGCGA GTACAACCTT	207	[29]
	ACTB_B	CCCATACCCACCATCACACC		
Glyceraldehyd-3-phosphate (*Gapdh*)	GAPDH2_A	CAAGTTCAACGGCACAGTCAAG	123	[30]
	GAPDH2_B	ACATACTCAGCACCAGCATCAC		
Mucin 2 (*Muc2*)	MUC2_A	TCCCTCTTACAAGGGCAATG	123	[31]
	MUC2_B	TTCCAGCTGTTCCCAAAGTC		
Claudin-1	CLDN-1_A	TGTCCACCATTGGCATGAAG	118	[32]
	CLDN-1_B	GCCACTAATGTCGCCAGACC		
Occludin	OCLN_A	GCCTTTTGCTTCATCGCTTC	125	[30]
	OCLN_B	AACACCATGATGCCCAGGAT		
Zonula occludens-1 (ZO-1)	ZO-1_A	AAGCCAGTCACGATCTCCCG	106	[30]
	ZO-1_B	GCGCTCTTCCTCTCTGCTCC		

### Animals and housing

6 week-old male Wistar rats were purchased from Taconic (Lille Skensved, Denmark) and originated from the same stable where faecal samples used to isolate TR2_39 were collected. On arrival the animals were housed in pairs and had *ad libitum* access to chow (Altromin 1324) and drinking water throughout the experiment. The environment was controlled with 12-hour light/dark cycles, temperature at 22 ± 1°C, relative humidity at 55 ± 5% and 8–10 air changes per hour. Animals were observed twice a day. Animal experiments were carried out at the National Food Institute, Technical University of Denmark (Mørkhøj facilities). Ethical approval was given by the Danish Animal Experiments Inspectorate (authorization number 2012-15-2934-00089). The experiments were overseen by the National Food Institutes in-house Animal Welfare Committee.

Four days after arrival the animals were weighed and cages were allocated randomly to the three experimental groups, namely CON (dosed with sterile water), XOS (dosed with XOS), and BIF (dosed with *B. pseudolongum* TR2_39) with 16 animals (8 cages) in each group. The XOS was obtained from Shandong Longlive Bio-Technology CO. Ltd, China as 95% pure powder extracted from corncob (zea). To limit potential effects of co-housing and coprophagia on the gut microbial composition, the animals were housed together for additionally 2 weeks before the dosing period was initiated. During the acclimatization period the weight of the animals, and the water and feed intake was monitored as intake per cage per day.

During the intervention period the animals were given oral gavage with 1 ml milliQ water (CON), 2 ml 500 mg/ml XOS (XOS) or 1 ml *B. pseudolungum* TR2_39, approximately 2.2-6.2*10^8^ CFU/ml (BIF) every second day for 14–16 days. The inoculum was prepared fresh for each dosing day from one aliquot of glycerol-frozen TR2_39, by anaerobic cultivation in four tubes with 45 ml BSM broth for approximately 48 hours followed by wash in reduced PBS and resuspension in PBS. The optical density was adjusted to OD_600_ = 10. Half of the animals were euthanized (CO_2_ chamber and decapitation) on day 14 and the remaining on day 16 after the initial dosing. Animals in the same cage were euthainised sequentially. Weight, water, and feed intake was monitored during the intervention period, as described for the acclimation period. Faecal samples were collected on Day 0 prior to initial dosage, and the day before euthanisation (Day 13 or 15) by collecting defecate directly in tubes. Samples were stored at -80°C until analysis.

### *In vivo*intestinal permeability assay

On the day of euthanisation, intestinal integrity was determined by measuring the permeability of FITC-dextran, using a similar approach as previously described [9]. Animals were fasted for at least 9 hours before the assay. From each cage, one animal was orally dosed with 0.5 ml 120 mg/ml FITC-dextran (4 kDa, Sigma-aldrich FD-4) per 100 g (corresponding to 600 mg/kg animal) bodyweight while the other was dosed with 0.5 ml PBS per 100 g bodyweight. Two hours after dosage, animals were euthanized and blood was collected from the neck directly into 50 ml Falcon tubes with 100 μl EDTA (0.5 M, pH 8, Ambion). Blood samples were immediately centrifuged (3800 rpm, 5 min) to collect plasma. Plasma was centrifuged again, diluted 1:1 in PBS and stored at 5°C until analysis on the same day. Analysis of each sample was done in triplicate by transferring volumes of 60 μl plasma-PBS solution to a black 96-well microtiter plate (Proxiplate-96 F, Perkin Elmer) and measuring the florescence at excitation 485 nm/emission 535 nm (Victor TM X4, Perkin Elmer). Standard curves were prepared for each of the euthanisation days, by adding fixed concentrations of FITC-dextran to plasma-PBS prepared from animals dosed with PBS.

### Dissection of animals

Only animals not dosed with FITC-dextran were dissected to exclude potential effects of FITC-dextran in the down-stream analysis. Abdomens were rinsed in 70% ethanol and dried with a paper towel before the incision. Approximately 2.5-4 cm from the caecum, an ileal section (0.5-1.0 cm) was removed and rinsed in PBS before storage in 1 ml RNAlater® (Life Technologies). Colonic sections were taken where the first pellet of content was visible (often 4–5 cm from caecum), and treated the same way as ileal samples. Finally, contents from the caecum were collected, where after the ceacal tissues were washed in PBS and stored in RNAlater®. Caecal contents were stored at -80°C, while tissues in RNAlater® were stored at 5°C overnight, and then transferred to -80°C.

### Collection of caecal content and caecal water

Caecal contents were weighed and homogenized 1:1 in MilliQ water. Slurries were centrifuged (11.000 g, 15 min) and the pellets stored at -80°C in aliquots of approximately 250 mg. Supernatants were centrifuged again and the pH was determined (Orion Star™ pH Benchtop Meter, Thermo Scientific) before sterile filtration (0.2 μm pore size, Sarstedt) and storage at -20°C.

### Extraction of bacterial DNA

DNA was extracted from faecal samples collected before the initial dosing (Day 0), the day before euthanisation (Day 13 or Day 15), as well as from caecal samples using the MoBio PowerLyzer® PowerSoil® DNA isolation kit (Mobio) following the recommendations of the manufacturer. DNA concentrations were determined using Qubit ds DNA HS assay kit (Invitrogen). DNA was stored at -20°C until further analysis.

### Ion Torrent sequencing

The bacterial composition was determined by sequencing of the V3-region of the 16S rRNA gene in bacterial DNA extracted from caecal contents, and from faecal samples collected before (Day 0) and after the intervention (Day 13 and Day 15) originating from animals not used for the FITC-dextran permeability assay (i.e. total of 24 animals). Amplification of the V3-region and subsequent sequencing was performed using the Ion Torrent PGM platform essentially as previously published [33]. Briefly, the V3-region of the 16S rRNA gene was amplified using a universal forward primer (PBU 5’-A-adapter-TCAG-barcode-CCTACGGGAGGCAGCAG-3’) with a unique 10–12 bp barcode for each bacterial community (IonXpress barcode as suggested by the supplier, Life Technologies) and a universal reverse primer (PBR 5’-trP1-adapter-ATTACCGCGGCTGCTGG-3’). PCR reactions were conducted with 4 μl HF-buffer, 0.4 μl dNTP (10 mM of each base), 1 μM forward primer, 1 μM reverse primer, 5 ng template DNA, and 0.2 μl Phusion High-Fidelity DNA polymerase (Thermo Scientific) in a reaction volume of 20 μl. Reactions were run at 98°C for 30 seconds followed by 24 cycles of 98°C for 15 seconds and 72°C for 30 seconds, before 72°C for 5 minutes and cooling at 4°C. Products were separated on a 1.5% agarose gel with SYBR-safe at 100 V for 90 minutes, visualized with the Safe Imager™ 2.0 (Invitrogen) and bands of expected size (approximately 260 bp) were excised from the gel. DNA was extracted using MinElute Gel extraction kit (Qiagen) following the recommendations of the manufacturer. DNA concentrations were determined with Qubit HS assay and a library constructed by mixing an equal amount of PCR products from each original community. Sequencing was performed on a 318-chip for Ion Torrent sequencing using the Ion OneTouch™ 200 Template Kit v2 DL. Sequence data were obtained in FASTQ format and further processed using CLC bio genomic workbench (Qiagen) in order to de-multiplex and remove sequencing primers. Further quality trimming using default settings (quality score = 0.05, trim ambiguous nucleotides = 2) and selection of reads with a final length between 110 bp – 180 bp was performed before exporting reads in FASTA format. The number of good quality reads used for taxonomical assignment ranged from 46,877 to 100,000. All sequence reads were taxonomically classified using the Ribosomal Database Project Multiclassifier tool [34]. A bootstrap cut-off ≥ 50%, was chosen as recommended for fragments below 250 bp and previously shown to be effective [35]. Relative abundance of bacterial taxa (family level) were determined for each community by comparing the number of reads assigned to a specific family to total number of reads assigned to the bacterial root. To limit variation between animals, the fold-change during the intervention was determined by calculating relative abundance before divided by relative abundance after, and log 2 transformations of these data. Bacterial taxa that were detected either before or after the intervention, but not in the corresponding before/after-sample from the same animal were set to 0.0005% analogous to 1 read in 200,000 reads.

### Quantitative PCR

The relative abundances of *Bifidobacterium* spp., *Lactobacillus* spp., and *Akkermansia muciniphila* in faecal samples from all animals as well as caecal samples were determined using quantitative PCR in a total reaction volume of 11 μl in 384-well microtiter plates using a LightCycler 480 II (Roche Applied Science). Each reaction contained 1X SYBR green mix (Roche Applied Science), 0,2 pmol/μl of each primer (Table 1), and 2 μl template DNA (1 ng/μl) and setup in four technical replicates with DNA from faecal samples collected before and after the intervention run on the same plate. Reaction conditions were: 95°C for 5 min, 40 cycles of 95°C for 10 sec, 60°C for 15 sec, and 72°C for 45 sec, followed by melting curve generation (95°C for 5 sec, 65 for 1 min and increasing the temperature to 98°C with a rate of 0.11°C/sec with continuous fluorescence detection). Data was initially analysed in the LightCycler® 480 software. Noise band and threshold was set automatically using the LightCycler® 480 software. Average C_q_-values of the four technical replicates calculated by the software were used for data analysis. Single C_q_ values differing by more than 2 cycles were considered outliers. The relative abundances of each gene target normalized to the total number of 16S rRNA genes (universal bacterial primer) were calculated as (1 + E_universal_)^Cq _ universal^/(1 + E_target_)^Cq _ target^_._ Mean PCR efficiency (E) for each primer set was calculated by use of the LinRegPCR software [36]. If the relative abundance was calculated to be below 0.001% of the total bacteria (corresponding to the ratio being below 10^-5^), it was set to half this value.

### RNA extraction and cDNA preparation

Total RNA was extracted from approximately 20 mg of ileum, caecum, and colon tissue using the RNeasy mini kit (Qiagen) following the suppliers recommendations. RNA concentration and purity was determined using Nanodrop Spectrophotometer ND-1000 (Thermo Scientific). Samples with A260/A280 between 1.8 and 2.1 were used in the further analysis. RNA was stored at -80°C. The cDNA was prepared immediately from 500 ng RNA in 20 μl reactions using the SuperScript VILO cDNA Synthesis Kit (Life technologies) following the suppliers recommendations and stored at -20°C until further use.

### Gene expression analysis

The relative gene expression of the tight junction proteins claudin-1, ZO-1, and occludin, as well as Mucin 2 (*Muc2*), involved in mucin production, were determined with quantitative PCR using actin beta (*Actb*) and glyceraldehyde 3-phosphate dehydrogenase (*Gapdh*) as reference genes (Table 1). Reaction conditions were as above and the reactions run under the following conditions; 95°C for 5 min, 40 cycles of 95°C for 10 sec, 60°C for 10 sec, and 72°C for 30 sec, followed by melting curve preparation 95°C for 5 sec, 65 for 1 min and 98°C continually. As template, 2 μl 10-fold diluted cDNA was used. The relative expression was calculated using the geometric mean of the two reference genes.

### Trans-epithelial resistance

The mammalian cell line Caco-2 (passage 15–25) were cultured in DMEM (Gibco) supplemented with 20% heat inactivated fetal bovine serum (Gibco), 1X Non-essential amino acids (Thermo Scientific), and 1X Pen/strep (Biological industries) at 37°C and 5% CO_2_. Cells were trypsinized when 60-80% confluent. A cell suspension of 10^5^ cells/ml was prepared and 500 μl was seeded in the apical compartment of 12 mm, 0.4 μm pore size Transwell® polyester membrane inserts (Corning, USA), while 1.5 ml medium was added to the basolateral compartment. Cells were cultured on the inserts for 21 days with change of medium twice a week. At day 21 the cells were moved to the cellZscope® (nanoAnalytics, Germany). Culture medium was changed, and 760 μl and 1.65 ml medium was added to the apical and basolateral compartment, respectively. TER was monitored for 20–23 hours. 76 μl medium was then replaced with caecal water, sterile milliQ water (control of the dilution of the cell culture media), or standard cell culture media (cell media control) (control of the cells), resulting in exposure to 5% caecal water. TER was subsequently measured every hour for 24 hours. All treatments were conducted in three replicates. All caecal water samples obtained from a given animal were analysed on the same day. Caecal water from the animals were used randomly, and placed randomly in the cellZscope®. The percentage changes in TER were determined based on the last measured TER before exposing the cells (t = 0). In most cases an average of the three replicates was calculated; however for a few samples only two replicates were used.

### Statistics

All data analysis was conducted in GraphPad Prims version 5.0 for Windows (GraphPad Software, CA, USA.) if not otherwise stated. Differences in animal weight, water intake, feed intake, FITC-dextran plasma concentrations, and caecal water pH between groups were assessed by one-way ANOVA with Bonferroni post-test or Kruskal-Wallis Dunns post-test for non-normally distributed data. The Metastats tool [37] was used for 16S rRNA gene sequence analysis using non-parametric t-tests based on 1000 permutations and setting the false discovery rate q = 0.05 as significant. For selected bacterial groups the relative abundances and fold-changes, determined by both 16S rRNA gene sequencing and qPCR, were also compared between CON and both XOS and BIF using Mann–Whitney *U*-test. Log 2 transformed fold changes were compared to a hypothetical median of zero using the Wilcoxon signed rank test. Differences in gene expression of tight junction proteins and *Muc2* between different types of tissue were determined for the CON group by one-way ANOVA with Bonferroni post-test or Kruskal Wallis test with Dunns post-test (not normally distributed data). Differences between CON and XOS or BIF for the individual tissues were determined using Mann–Whitney *U*-test. Correlation analysis was determined using the Spearman correlation, considering P < 0.05 to be significant. The Χ^2^-test was used to compare the number of observed differences between faecal and caecal samples in the three groups.

## Results

### Animal growth, feed, and water intake

**Figure 1:**
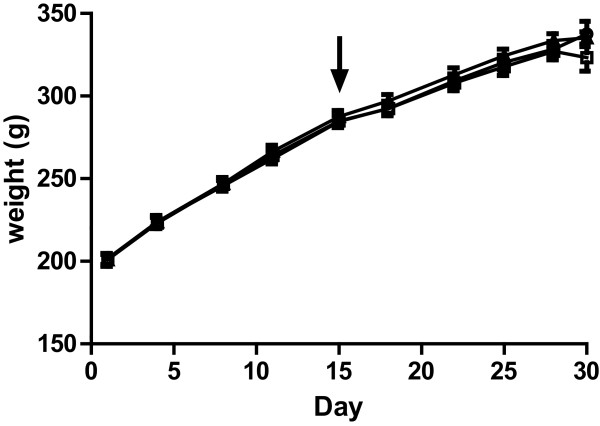
**Animal weight gain during the study.** Mean with SD is illustrated for each treatment group; CON (circles), XOS (triangles), and BIF (squares). The arrow indicates initiation of the dosing period.

There were no significant differences in animal weight gain between the three groups (Figure 1). Additionally, no significant differences in water and feed intake between the three groups were recorded (data not shown).

### Bacterial composition

**Figure 2:**
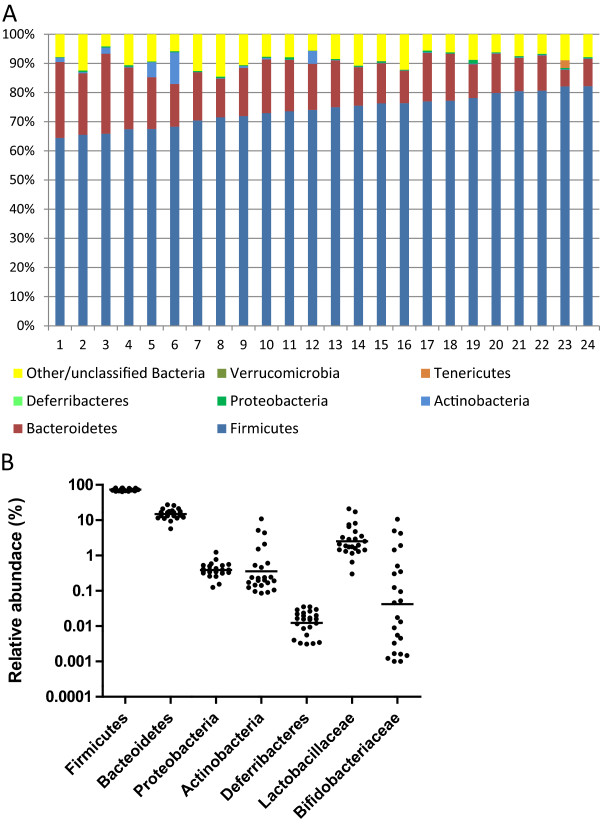
**Bacterial community composition of individual animals before intervention based on 16S rRNA gene sequencing. A**: Bacterial community composition at phylum level for one animal from each of the 24 separate cages. Columns are ordered with increasing relative abundance of *Firmicutes*. **B**: The relative abundance for selected phyla and families are shown as dot-plots with geometric average indicated by a horizontal line.

**Figure 3:**
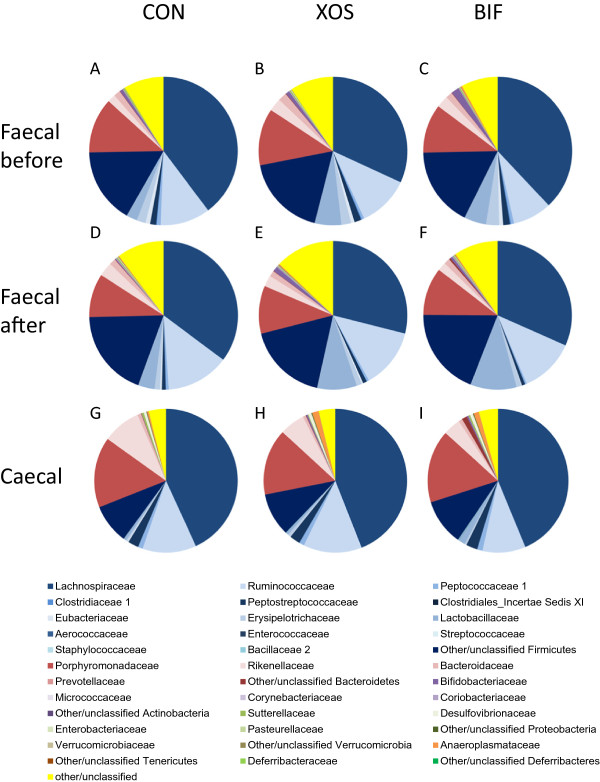
**Bacterial community composition in faecal and caecum content samples based on 16S rRNA gene sequencing.** The mean bacterial composition is shown at the family level for faecal samples obtained before intervention **(A-C)**, after intervention **(D-F)** and caecal content samples **(G-I)** for animal in CON, XOS and BIF groups. Differences in mean relative abundances were only observed between faecal and caecal samples as detailed in Table 2.

Bacterial community analysis at phylum level based on 16S rRNA sequencing of faecal samples from 24 animals (one from each cage) before intervention revealed variation in the relative abundance (Figure 2A), and markedly *Actinobacteria* varied approximately 100-fold from 0.085% to 10.9% and *Bifidobacteriaceae* 10,000-fold from 0.001% to 10.7% between individual animals (Figure 2B). Significant negative correlations were found between *Bacteroidetes* and *Firmicutes* (P < 0.0001, R = -0.82) and *Firmicutes* and *Actinobacteria* (P = 0.019, R = -0.48) and also a negative correlation between *Bacteroidetes* and *Actinobacteria* (P = 0.023, R = 0.46). No significant differences in relative abundances before and after intervention were found between any of the detected bacterial families in faecal samples from the two intervention groups as compared to the CON group after correction for multiple testing (Figure 3). Neither did principal component analysis of sequencing data at the family-level show any clustering of samples according to intervention group (data not shown). Additionally, no differences in the fold-change (after/before) of any of the detected bacterial families were found between the groups after correction for multiple testing (data not shown). We did however observe differences in the mean relative abundances of several bacterial families between faecal samples and caecal content samples (Table 2).

**Table 2:** **Differences detected between caecal and faecal samples**

Phylum	Family	Sample	CON	XOS	BIF
			Mean ± SEM	Mean ± SEM	Mean ± SEM
*Firmicutes*	*Lachnospiraceae*	Faecal	3.6E-01 ± 3.6E-02	**2.9E-01 ± 3.7E-02**	3.2E-01 ± 4.5E-02
		Caecal	4.3E-01 ± 4.4E-02	**4.5E-01 ± 3.0E-02**	4.4E-01 ± 2.8E-02
	*Peptostreptococcaceae*	Faecal	**7.7E-03 ± 2.2E-03**	**8.2E-03 ± 3.0E-03**	**7.0E-03 ± 2.0E-03**
		Caecal	**2.4E-02 ± 8.7E-03**	**2.4E-02 ± 4.9E-03**	**2.6E-02 ± 5.0E-03**
	*Erysipelotrichaceae*	Faecal	1.2E-02 ± 3.6E-03	1.1E-02 ± 2.9E-03	**1.1E-02 ± 2.5E-03**
		Caecal	3.9E-03 ± 8.9E-04	6.1E-03 ± 2.7E-03	**3.8E-03 ± 6.3E-04**
	*Lactobacillaceae*	Faecal	**3.4E-02 ± 8.0E-03**	**8.5E-02 ± 2.7E-02**	9.7E-02 ± 3.4E-02
		Caecal	**5.9E-03 ± 3.4E-03**	**6.0E-03 ± 8.0E-04**	1.5E-02 ± 6.6E-03
	*Streptococcaceae*	Faecal	**3.6E-04 ± 6.9E-05**	3.8E-04 ± 1.1E-04	2.2E-04 ± 5.2E-05
		Caecal	**1.3E-04 ± 3.6E-05**	2.1E-04 ± 8.3E-05	1.9E-04 ± 7.1E-05
	*Staphylococcaceae*	Faecal	**1.2E-04 ± 3.3E-05**	8.0E-05 ± 1.2E-05	1.2E-04 ± 1.4E-05
		Caecal	**3.6E-05 ± 9.0E-06**	4.0E-05 ± 1.1E-05	6.2E-05 ± 2.2E-05
	*Veillonellaceae*	Faecal	**N.D.**	**N.D.**	**N.D.**
		Caecal	**2.6E-04 ± 1.6E-04**	**9.5E-04 ± 7.5E-04**	**4.8E-04 ± 3.2E-04**
*Bacteroidetes*	*Rikenellaceae*	Faecal	**3.0E-02 ± 5.6E-03**	2.4E-02 ± 5.7E-03	1.9E-02 ± 4.6E-03
		Caecal	**8.6E-02 ± 2.1E-02**	5.3E-02 ± 1.2E-02	4.0E-02 ± 9.4E-03
*Actinobacteria*	*Micrococcaceae*	Faecal	**2.6E-04 ± 5.4E-05**	**1.4E-04 ± 3.1E-05**	**1.7E-04 ± 2.3E-05**
		Caecal	**3.1E-05 ± 1.1E-05**	**2.8E-05 ± 1.0E-05**	**6.5E-05 ± 1.7E-05**
	*Corynebacteriaceae*	Faecal	**6.6E-05 ± 1.5E-05**	**5.9E-05 ± 2.0E-05**	5.4E-05 ± 1.1E-05
		Caecal	**1.9E-05 ± 6.3E-06**	**1.0E-05 ± 4.6E-06**	5.8E-05 ± 3.9E-05
	*Coriobacteriaceae*	Faecal	**1.5E-03 ± 3.2E-04**	**1.7E-03 ± 1.9E-04**	1.9E-03 ± 4.6E-04
		Caecal	**2.4E-04 ± 6.1E-05**	**2.5E-04 ± 4.8E-05**	5.5E-04 ± 1.4E-04
*Proteobacteria*	*Desulfovibrionaceae*	Faecal	**1.3E-03 ± 4.9E-04**	9.9E-04 ± 5.6E-04	1.1E-03 ± 5.6E-04
		Caecal	**5.9E-03 ± 1.1E-03**	5.7E-03 ± 3.1E-03	6.5E-03 ± 2.2E-03
	*Hyphomicrobiaceae	Faecal	**N.D.**	**N.D.**	1.9E-06 ± 1.9E-06
		Caecal	**3.2E-05 ± 9.8E-06**	**4.2E-05 ± 1.9E-05**	3.9E-05 ± 2.1E-05
*Deferribacteres*	*Deferribacteraceae*	Faecal	**1.0E-04 ± 2.4E-05**	1.3E-04 ± 6.0E-05	1.1E-04 ± 2.0E-05
		Caecal	**4.1E-04 ± 9.2E-05**	4.1E-04 ± 1.3E-04	3.5E-04 ± 8.3E-05

Mean ± SEM are shown and highlighted in boldface for those families with significant differences after correction for False Discovery Rate (q < 0.05).

*Note that the family *Hyphomicrobiaceae* contains the genera *Gemmiger*, which shows high 16S rRNA gene sequence homology to members of the *Ruminococcaceae* family (*Firmicutes*), and may thus be taxonomically misplaced.

**Figure 4:**
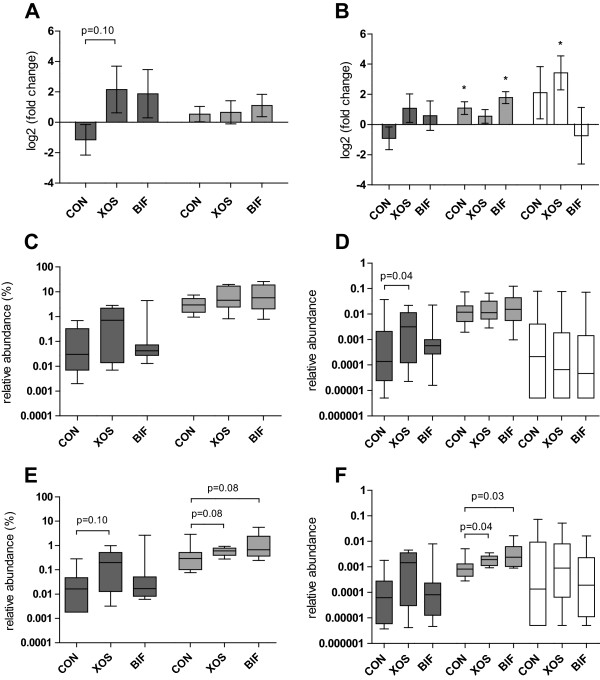
**Fold changes and relative abundances determined by 16S rRNA gene sequencing and qPCR.** Columns show means with SEM **(A-B)** or box and whisker plots with full range **(C-F)** for *Bifidobacteriaceae* (dark grey) and *Lactobacillacae* (light gray) determined by high through-put sequencing **(A,C & E)** and for *Bifidobacterium* spp. (dark grey), *Lactobacillus* spp. (light gray) and *Akkermansia muciniphilla* (white), determined by qPCR **(B,D and F)**. *Akkermansia muciniphilla* was not included in 16S sequencing due to low abundance. Analysis were performed on community DNA extracted from faecal samples **(A-D)** or caecum content **(E-F)**. In panels **A** and **B**, significant differences from baseline are indicated with asterisks (p < 0.05). Observed differences between groups are indicated with P-values.

Analyses of relative abundance and fold-change during the intervention for bacteria belonging to the *Bifidobacteriaceae* and *Lactobacilliaceae* were conducted separately as we hypothesized these groups to be affected and also included qPCR-based assessment of the relative abundance of *Bifidobacterium* spp., *Lactobacillus* spp., and *Akkermansia muciniphila* (Figure 4). Taken together, results obtained by qPCR (Figure 4B, D, and F) appeared very similar to the sequencing data (Figure 4A, C, and E). Fold-change data show that *Lactobacillus* spp. increased in the CON group (P = 0.014) and the BIF group (P = 0.0018) compared to baseline (qPCR data). In addition, *A. muciniphila* significantly increased compared to baseline in the XOS intervention group (P = 0.014). There were no significant differences in fold-change for either of the bacterial taxa between the control and the two treatment groups. Sequencing data revealed a trend for a larger fold-change of *Bifidobacterium* spp. in the XOS group than in the CON group (P = 0.10), however this was not confirmed by qPCR (P = 0.19). Nevertheless, qPCR showed that the relative abundance of *Bifidobacterium* spp. in faeces (Figure 4D) was higher in the XOS group than in the CON group (P = 0.044), while this was not confirmed by sequencing data (Figure 4C, P = 0.23).

In caecal content (Figure 4E and F) both the XOS and BIF groups had higher relative abundance of *Lactobacillus* spp. than the CON group (XOS; P = 0.04, BIF; P = 0.03) according to qPCR, while a tendency for this was confirmed by sequencing analysis (XOS; P = 0.08, BIF; P = 0.08). Additionally, XOS tended to increase *Bifidobacteriacae* in caecum content (P = 0.10) detected by sequencing.

### Intestinal permeability

**Figure 5:**
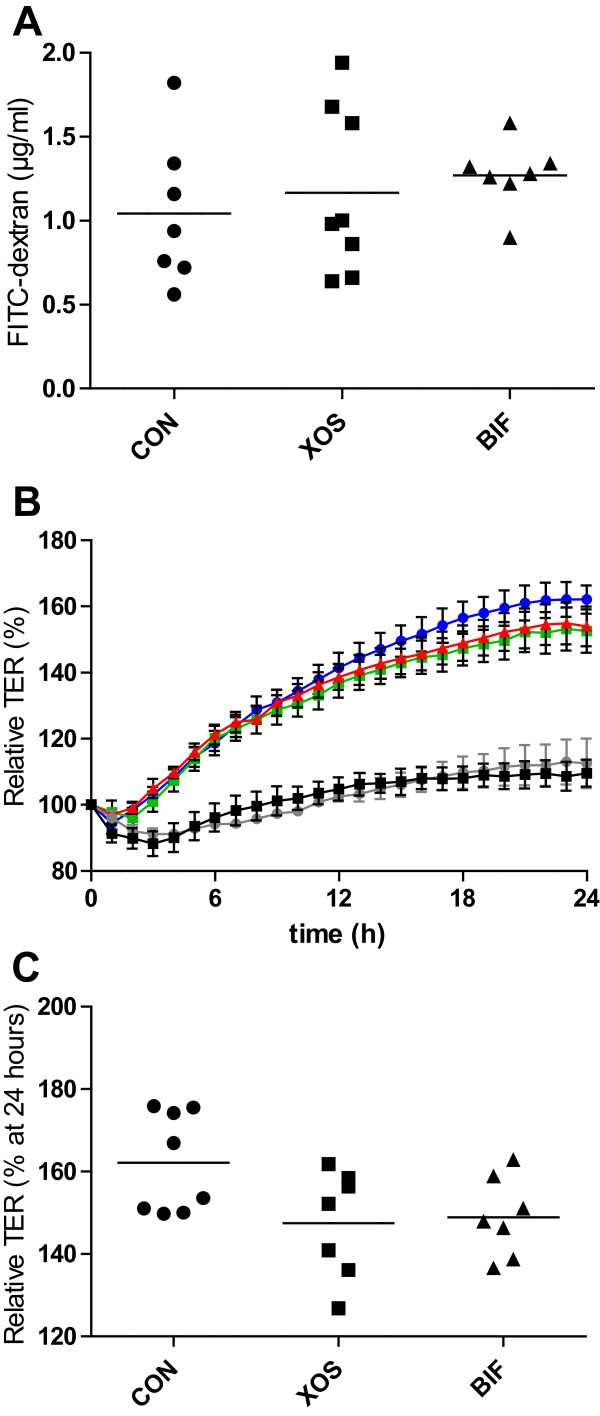
**Gut integrity as determined by FITC-dextran permeability and Trans-Epithelial Resistance (TER).** FITC-dextran concentrations in plasma **(A)** and relative TER across Caco-2 cells exposed to caecal water during the 24-hour exposure period **(B)** and at 24 hours after exposure **(C)**. Dot plots with means indicated by horizontal lines **(A and C)** and mean values with SEM for groups CON (blue circles), XOS (green squares), BIF (red triangles) as well as water (grey circle) and cell media control (black squares) are shown **(B)**.

No differences in FITC-dextran concentration in the plasma were observed between the three groups (Figure 5A). The results from two animals, one from the CON group, and one from the BIF group, were excluded due to technical errors.

The average caecal water pH was 7.53 ± 0.15 (SD), 7.48 ± 0.23, and 7.58 ± 0.20 for the XOS group, BIF group, and CON group, respectively with no significant differences between the groups. Caecal water from all three groups on average significantly increased TER as compared to the controls exposed to water or pure cell media (Figure 5B), but no significant differences were found between the three experimental groups after 24 hours of exposure (Figure 5C), although the TER was consistently lower in all time points between 12 and 24 hours after exposure to caecal water from either of the treatment groups as compared to CON (Figure 5B).

### Gene expression

**Figure 6:**
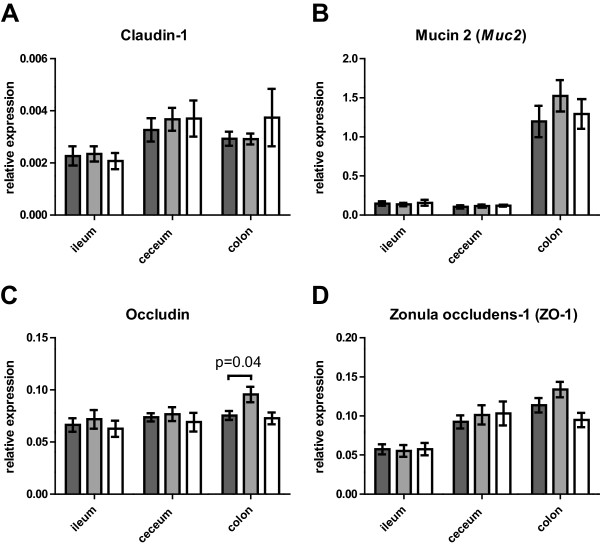
**Gene expression of intestinal permeability markers.** Mean relative gene expression of claudin-1 **(A)**, *Muc2* **(B)**, occludin **(C)**, and ZO-1 **(D)** in tissue samples obtained from ileum, caecum and colon from animals in CON (dark gray), XOS (light gray) and BIF (white) groups are shown. Observed differences between groups are indicated with p-values. Error bars indicate SEM.

Differences in gene expression between tissue types were determined for the CON group (Figure 6). Expression of *Muc2* was higher in the colonic tissue than in ileal (P < 0.01) and caecal tissue (P < 0.001), and also expression of ZO-1 was higher in colon than ileum (P < 0.001). The expression of claudin-1 and occludin did not differ between the intestinal sections. The relative expression of occludin in colon was higher (P = 0.04) in the XOS group than in the CON group (Figure 6C). No other significant differences between the groups were found.

### Correlations between gene expression of epithelial cells, measures of intestinal integrity and relative abundance of selected bacterial groups

No significant correlations were found between the relative abundance of *Bifidobacterium* spp., *Lactobacillus* spp. or *Akkermansia muciniphilla* in caecal content and faecal samples (qPCR), and relative gene expression of claudin-1, ZO-1, *Muc2*, and occludin in ileal, caecal and colonic tissue, as well as plasma FITC-dextran concentrations and TER, irrespective of experimental group (data not shown).

## Discussion

Changes in the gut microbial composition have been proposed to affect intestinal integrity [9]. The present study was designed to address this issue further by focusing on the effects of bifidobacterial abundance on microbial community composition and intestinal integrity in male Wistar rats. Two different approaches were used to increase levels of bifidobacteria, namely (i) oral dosage with live cultures of an endogenously isolated strain (probiotic approach) and (ii) oral dosage with XOS, which has previously been shown to stimulate bifidobacterial growth in a mouse model [17] (prebiotic approach).

Experimental animals bred and treated under standardized conditions are generally expected to exhibit less inter-individual variation than a free-living human population and consequently it should require fewer individuals to find effects in dietary intervention studies. Comparison of the animals at base-line (Figure 2A) revealed less variation within the two most abundant phyla, *Firmicutes* and *Bacteroidetes*, than reported in human studies [38], but interestingly, for bacteria belonging to the *Actinobacteria*, a more than 100-fold difference in relative abundance was observed between animals. For the *Bifidobacteriaceae* family, belonging to the *Actinobacteria*, we observed approximately 10,000-fold difference in relative abundance before the intervention commenced (Figure 2B). The high initial level of variation within the *Bifidobacteriaceae* in this study may impede detection of the expected XOS or BIF driven increase in relative abundance of this bacterial group during the intervention, as such an increase was only detectable by qPCR, and not by sequencing of community-derived 16S genes. The increased relative abundance of bifidobacteria following intake of XOS is consistent with a previous study in male Sprague–Dawley rats, which showed increase in both faecal and caecal levels of bifidobacteria following a 14-day intervention with XOS added to feed at 6% [39] and also an increase is reported in XOS-fed mice [17]. Animals in the BIF group received approximately 2.2-6.2*10^8^*B. pseudolungum* cells every second day during the intervention. This did however not result in higher levels of bifidobacteria in either caecum content or faecal samples at termination. In spite of the fact that the bifidobacterial strain applied was isolated from similar rats, we speculate that the strain did not colonize and/or proliferate in the rat gut, resulting in washout before faecal samples were obtained approximately 24 hours after the last dosage. A study addressing intestinal transit of *B. bifidum* following gavage in mice showed a peak in the abundance of this strain in faeces at around 6 hours after dosage and subsequently a significant reduction after 18 hours [40]. Alternatively, the dosing level was too low to have an effect or bifidobacterial cells may not have survived passage through the acidic environment of the rat stomach.

Quantitative PCR as well as 16S rRNA amplicon sequencing revealed higher caecal levels of *Lactobacillus* spp. in both the XOS and BIF groups compared to the CON groups after intervention (Figure 4E-F). This is consistent with a prebiotic effect of XOS [6] and confirms that increasing the abundance of one bacterial group may influence the abundance of another through e.g. metabolic cross-feeding processes [41] or by changing environmental conditions such as pH. Detection of significant differences in the relative abundance of *Lactobacillus* spp. between the groups was facilitated by a relatively low initial variation of *Lactobacilliaceae* (approximately 70-fold) compared to *Bifidobacteriaceae* (Figure 2B). Quantatative PCR is anticipated to result in better quantification than amplicon sequencing, especially for low-abundant bacterial groups, due to the low absolute number of sequence reads in the latter. In the present study we observe only slightly more significant differences by the qPCR approach compared to the sequencing approach (Figure 4) indicating only marginally higher power.

The mucin degrading species *A. muciniphila* was included in the qPCR analysis, due to its status as potential marker for intestinal health (reviewed by [42]). An increase in levels of *A. muciniphila* after the intervention compared to baseline was found only in the XOS group (Figure 4B). This may be explained by a XOS-induced increased production of mucin, as *A. muciniphila* is capable of degrading mucin as sole carbon source [43]. Also *A. muciniphila* is reported to be reduced in patients suffering from disruption of the gut mucus layer due to mucosal inflammation [44] as well as in ob/ob mice [45]. Prebiotics have previously been shown to normalize, hence increase, *A. muciniphila* abundance in obese and type 2 diabetic mice and also administration of viable *A. muciniphila* was connected to improvement of metabolic disorders in mice fed a high-fat diet, potentially due to reestablishment of the mucus layer [45]. Nevertheless, we observed no differences in expression of the mucin gene (*Muc2*) between the three experimental groups in any of the intestinal segments (Figure 6B). However, as the actual amount of mucus was not determined, this does not exclude the possibility of increased mucin levels in the XOS group due to post-transcriptional alterations and/or increased expression of other mucin encoding genes. Previously increased levels of mucin secretion were reported in animals fed FOS [14, 18, 46]. Mucins secretion was also increased in humans, but this was not connected to altered permeability for CrEDTA [47].

The overall mean gut microbiota composition in faecal samples was very similar in all three groups before the intervention and remained so during the intervention (Figure 3). No differences in microbiota composition after the interventions were observed between treatment groups after correction for multiple comparisons (Figure 3). We observed several bacterial families which differed in mean relative abundance in caecum content compared to faecal samples, including higher levels of *Actinobacteria* and lower levels of *Peptostreptococcaceae* and *Veillonellaceae* associated with faecal samples in all three intervention groups (Table 2). We observed fewer families that differed in relative abundance between faeces and caecum content in the XOS and BIF groups than in the control group but this was not significant (Χ^2^-test).

Measures of rat gut integrity were obtained by three independent measures namely (i) permeability of FITC-dextran molecules across the epithelial barrier (Figure 5A), (ii) trans-epithelial resistance of Caco-2 cells after exposure to caecal water (Figure 5B-C), and (iii) relative expression of genes encoding tight junctions proteins or mucin (Figure 6). These measures were selected to collectively cover different aspects of gut permeability. Intestinal permeability is mainly determined by paracellular transport between epithelial cells, which has been suggested to be divided into two pathways: The high-capacity “pore pathway” where small molecules (below 4 Å) can pass, and the low-capacity “leak pathway” where larger molecules may pass (reviewed by [48]). Changes in FITC-dextran permeability indicate a change in the leak-pathway, while changes in TER may indicate changes in both pathways [48]. We found no statistically significant effect on either FITC-dextran permeability or TER after 24 hours between treatment groups and the CON group of animals (Figure 5A and C). Nevertheless, TER was observed to be consistently higher in the CON than both the XOS and BIF groups from around 12 hours until termination at 24 hours, indicating an increase in permeability in the Caco-2 monolayer during exposure to caecal water from XOS and BIF (Figure 5B). This is consistent with a previously observed trend for a negative correlation between TER and relative abundance of bifidobacteria [11]. Caecal-water collected from CON, XOS of BIF animals increased TER during 24-hours significantly more than water, which was used as control. This suggests that caecal water positively affects tight-junction interaction, which is consistent with similar observations on faecal-water [11]. Expression levels of occludin genes in colonic tissue were significantly higher in the XOS group than in the CON group. Changes in expression of ZO-1 and occludin in ob/ob mice after consumption of prebiotics have previously been studied showing that prebiotic treatment increased levels of *Bifidobacterium* spp. as well as occludin and ZO-1 expression in jejunum, and also decrease FITC-dextran (4 kDa) permeability [49]. Additionally, high-fat feeding was reported to decrease *Bifidobacterium* spp., increase intestinal permeability and decrease the expression of ZO-1 and occludin [9]. It should be noted that specific strains of bifidobacteria may have varying effects on markers of intestinal integrity [19, 50, 51], which could explain the relatively minor effect of the *B. pseudolongum* isolate in the current study.

## Conclusion

The present study was designed to address the hypothesis that increased levels of bifidobacteria are linked to decreased intestinal integrity caused by modulation of the microbiota, as indicated by previous studies showing increased *Salmonella* translocation following intake of prebiotics in rodents [14–17]. However, this hypothesis was not confirmed, perhaps because the limited effects of XOS and dosage of bifidobacteria on intestinal bifidobacterial loads were insufficient to induce measurable changes in intestinal integrity. Our observations of increased occludin expression after XOS consumption seem to contradict the hypothesis, while the consistent decrease in TER caused by caecal water from BIF and XOS rats, although not significant, points in a confirmatory direction.
